# Repurposing Carfilzomib as a Promising Drug for Targeted Therapy in Gastric Cancer

**DOI:** 10.3390/cancers17213420

**Published:** 2025-10-24

**Authors:** Emma Mathilde Kurstjens, Kristin E. Cox, Prerna Bali, Siamak Amirfakhri, Jonathan Hernandez, Ivonne Lozano-Pope, Christopher Benner, Michael Bouvet, Marygorret Obonyo

**Affiliations:** 1 Department of Medicine, School of Medicine, University of California San Diego, La Jolla, CA 92093, USA; 2 Moores Cancer Center, University of California San Diego, La Jolla, CA 92093, USA; 3 Department of Surgery, University of California San Diego, La Jolla, CA 92093, USA; 4 VA San Diego Healthcare System, San Diego, CA 92161, USA

**Keywords:** *PSMB8*, carfilzomib, targeted therapy, gastric cancer, *Helicobacter*

## Abstract

**Simple Summary:**

Gastric cancer (GC) is often detected at advanced stages, leading to a need for effective targeted therapies. In this study, we identified *Psmb8* as a putative therapeutic target based on gene expression profiling from our accelerated *Helicobacter*-induced gastric cancer mouse model. We observed elevated levels of *PSMB8* in both a human GC epithelial cell line and patient samples. Thereafter, we identified carfilzomib as a drug that selectively targets *PSMB8* and tested its efficacy on a human-cell-line-derived xenograft model. Carfilzomib inhibited tumor growth by inducing tumor cell loss via apoptosis and impeding cell proliferation. This suggests that it has robust anti-tumor activity and has potential to be used as a treatment for cancers where high levels of *PSMB8* are associated with poor overall survival.

**Abstract:**

**Background/Objectives**: Identifying novel targets to treat gastric cancer (GC) has become a focus of research in recent years. Our accelerated *Helicobacter*-induced gastric cancer mouse model allowed us to identify several differentially expressed genes (DEGs), including *Psmb8* (proteasome subunit beta type 8, also called Lmp7), which was also found to be elevated in GC patient samples. *PSMB8* encodes one of the immune subunits of the immunoproteasome, which has been associated with disease severity in multiple cancers. **Methods:** We identified carfilzomib from a public database as a potential drug targeting *PSMB8*; it effectively halts immunoproteasome activity, leading to apoptosis. We tested carfilzomib’s efficacy against gastric cancer by subcutaneously implanting nude mice with human gastric epithelial-derived tumors and treating them with carfilzomib, either alone or in combination with 5-fluorouracil (5-FU), a standard-of-care drug. The effectiveness of drug treatment was measured by tumor growth, cell proliferation, and apoptosis. **Results:** We observed that carfilzomib retarded tumor growth, inhibited cell proliferation, and induced apoptosis. **Conclusions:** These results strongly suggest that *PSMB8* is a suitable candidate for targeted therapy. Moreover, with carfilzomib having robust anti-tumor activity, it has potential as a treatment option for cancers where high levels of *PSMB8* are associated with poor overall survival.

## 1. Introduction

*Helicobacter pylori* (*H. pylori*) infection and consequent inflammation is a major risk factor leading to gastric cancer (GC). Based on a recent meta-analysis, *H. pylori* infects approximately 43.9% of adults and 35.1% of children and adolescents globally [1]. Though there are many risk factors that contribute to the development of GC, including Epstein–Barr virus (EBV) [2] and genetic inclination, infection with *H. pylori* is the main risk factor, and this bacterium is classified as a class I carcinogen [3,4]. GC is the fifth leading cause of cancer-related deaths, with an estimated 770,000 deaths reported in 2020 [5,6]. Moreover, *H. pylori* caused over a third of infection-induced cancers in 2020, surpassing human papillomavirus, as reported by the World Health Organization [7]. Despite the overwhelming prevalence of this bacterium and availability of genomic data, GC has a high mortality rate due to late diagnosis. The trend of late diagnosis is due to the non-specificity of common symptoms—dysphagia and weight loss—and their tendency to appear during the advance stages of disease [8,9]. Advanced stage diagnosis has a notable effect on the 5-year survival rate, which is 36.4% in the US [10]. Consequently, there is a medical need for novel and effective targeted therapies [8].

In recent years, targeted therapies for GC have become a focus of research. Since most cases of GC are diagnosed at stages III–IV, the standard procedure of care for operable malignancies consists of surgical resection and perioperative nonspecific chemotherapy regimens, such as FLOT (5-fluorouracil, leucovorin, oxaliplatin, and docetaxel) [8,9,11,12]. Perioperative application of therapies targeting markers, such as human epidermal growth factor receptor 2 (*HER2*), vascular endothelial growth factor (*VEGF*), claudin 18.2 (*CLDN18.2*), and programmed death-ligand 1 (*PDL-1*), have recently been applied in a clinical setting, with promising results [11,12]. Moreover, using the Prognostic Nutritional Index (PNI) tool preoperatively in GC patients has been reported to be helpful in predicting postoperative complications and the long-term survival of patients with GC [13]. However, more markers need to be identified in order to expand the range of patients who can receive targeted therapy, especially given that not all GC tumors express these above-listed markers. Tumor heterogeneity, a marked trait of GC, also plays a role in complicating biomarker expression, as it has been seen that the variation in the types of cells involved in gastric carcinogenesis is indicative of variation in gene expression [14,15,16]. Therefore, to address this gap in patient care, we reanalyzed data from our accelerated GC model [17,18,19] to identify potential therapy targets. Reanalysis of both microarray and RNA-seq data collected from mouse stomach tissue sections revealed several differentially expressed genes (DEGs) that were associated with severe disease pathology, such as indoleamine 2,3-dioxygenase 1 (*Ido1*), caspase 1 (*Casp1*), matrix metalloproteinase 13 (*Mmp13*), proteasome subunit beta 8 (*Pmsb8)*, proteasome subunit beta 9 (*Psmb9*), and proteasome subunit beta 10 (*Psmb10*) [17,19].

Given that these DEGs were associated with severe disease pathology, this led us to hypothesize that they may be involved in promoting *Helicobacter*-induced disease progression. Therefore, to test this hypothesis by targeting these DEGs, we first evaluated their expression in a human GC epithelial cell line (MKN45). We observed that *PSMB8* was the most highly expressed DEG. *PSMB8* is one of the immune subunits (β5i) of the immunoproteasome; it has a chymotrypsin-like activity [20] and its elevated expression in different cancers has been associated with poor disease outcome [1]. Therefore, for clinical relevance, we validated the expression of *PSMB8* in human GC patient tissue samples. Those findings led us to assess the potential of *PSMB8* as a drug target candidate for GC treatment.

Thereafter, we explored the publicly available pharmaceutical databases [21] and identified carfilzomib as a potential drug capable of targeting *PSMB8*. Carfilzomib has already been approved by the US Food and Drug Administration for the treatment of patients with relapsing or refractory multiple myeloma, both alone and in combination with dexamethasone [22,23]. Repurposing an already approved drug aids in developing a treatment regime faster than traditional drug development and is also more cost effective [22,23,24,25]. Carfilzomib is a second-generation proteasome inhibitor comprised of an N terminal morpholine, a tetrapeptide, and an epoxyketone that inhibits immunoproteasome activity by covalently binding the catalytic N-terminal threonine in the specificity pocket of the β5i subunit (*PSMB8*), halting its chymotrypsin like activity [26,27]. Disabling the immunoproteasome in this way results in an accumulation of polyubiquitinated proteins, initiating apoptosis via intrinsic and extrinsic pathways [26,27,28]. This binding is irreversible and specific at therapeutic concentrations.

In this study, we investigated the efficacy of carfilzomib in treatment of GC, either alone or in combination with 5-fluorouracil (5-FU)—a standard treatment drug—to assess its potential as a suitable candidate for drug repurposing to treat GC. In addition, we evaluated whether additive or synergistic effects could be achieved. We monitored tumor growth and assessed cell proliferation and apoptosis. The data in the present study indicate that carfilzomib hampered tumor growth and cell proliferation and induced apoptosis, making it a promising drug for targeted GC treatment.

## 2. Materials and Methods

### 2.1. Animals

All the animal procedures were approved by the University of California San Diego Institutional Animal Care and Use Committee (IACUC) and conducted following accepted veterinary standards and ARRIVE guidelines. Athymic male nude mice, aged 4–6 weeks, were used for the experiments. The animals were fed an autoclaved diet and housed in a barrier facility. Prior to any surgical procedure, the mice were anesthetized with a solution of xylazine, ketamine, and phosphate-buffered saline (PBS) via intraperitoneal injection. At the conclusion of the study, mice were anesthetized with isoflurane and euthanized by cervical dislocation to remove tumors.

### 2.2. Human Participants

Twenty-six de-identified snap-frozen human gastric biopsy samples were obtained from University of California San Diego Biorepository, Moores Cancer Center. Prior to sample collection, all patients provided written informed consent and were subsequently followed up. Details about the patients and their gastric tumor characteristics, including race or ethnicity and clinical data, are provided in Table 1. RNA was extracted from each biopsy tissue and subsequently processed for real-time PCR as described below. The study was conducted in accordance with the Declaration of Helsinki and approved by the Institutional Review Board of the University of California, San Diego.

### 2.3. Xenograft Establishment

**Figure 1:**
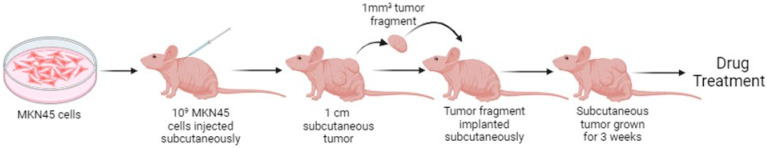
Schematic diagram showing the process of establishing the xenograft model in preparation for experimental treatment. Created with BioRender.com.

A human GC epithelial cell line, MKN45, was used for these experiments. Cells were cultured in RPMI media supplemented with 10% fetal bovine serum (FBS), penicillin (100 U/mL), and streptomycin (100 µg/mL). Initially, in order to establish subcutaneous models, 1 × 10^6^ MKN45 cells suspended in 100 µL of PBS were injected into the bilateral flanks and shoulders of nude mice. Once subcutaneous tumors had grown to approximately 1 cm, subsequent passages were performed by harvesting 1 mm^3^ fragments and implanting them into additional nude mice. For the experiments, a ~5 mm incision was made on the mid-back of the nude mice and a single 1 mm^3^ tumor fragment was implanted into the right flank. The incision was closed with a simple interrupted 6-0 vicryl suture (Ethicon Inc., Raritan, NJ, USA). Tumors were allowed to grow for 3 weeks before mice received any treatment. This protocol is summarized in Figure 1.

### 2.4. Treatment Regime

**Figure 2:**
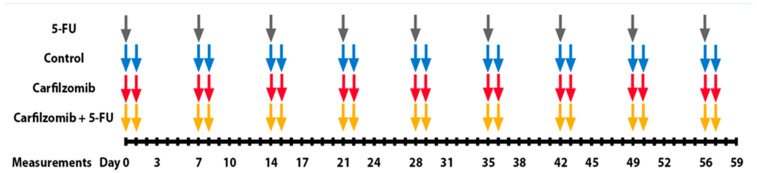
Schematic diagram showing the schedule of intraperitoneal injections delivered to experimental mice.

Nude mice were divided into 4 groups (*n* = 10/group)—5-FU (administered 5-FU only), carfilzomib (administered carfilzomib only), combination (administered both 5-FU and carfilzomib), and control (administered placebo control). 5-FU was purchased from Acros Organics BVBA and resuspended in PBS to make a stock solution of 10 mg/mL. Carfilzomib was identified using a public pharmaceutical database called the Drug Gene Interaction database (DGIdb) [21]. Carfilzomib was purchased from Onyx Pharmaceuticals and resuspended in 2 mL of dimethyl sulfoxide (DMSO) to make a stock solution of 50 mg/mL. 5-FU and carfilzomib were administered at dosages of 50 mg/kg and 5 mg/kg, respectively. PBS containing 2% DMSO served as the placebo control. Both drugs and the placebo control were administered via intraperitoneal (IP) injections at a total volume of 150 µL. One dose of 5-FU was administered per week for 8 weeks [29]. Carfilzomib and placebo control were administered for two consecutive days per week for 8 weeks [30]. The drug dosing schedule is summarized in Figure 2.

### 2.5. Animal Monitoring

**Figure A1:**
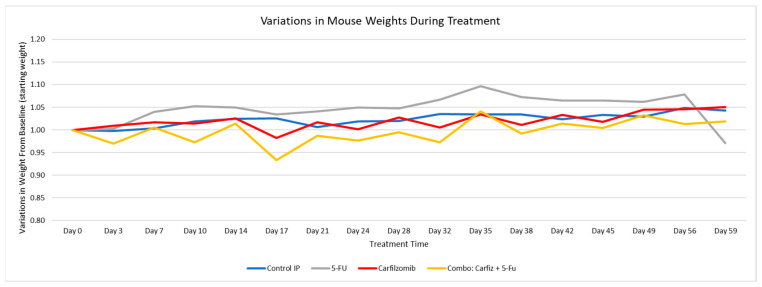
Variations in mouse weight graphed as changes from the baseline (beginning weight). Each line on the graph represents the average weights of individuals from each respective treatment group. Control group *n* = 6; 5FU group *n* = 8; Carfilzomib group *n* = 7; Combination *n* = 8.

Mice were monitored for both tumor growth and body weight twice a week. Tumor size was measured using calipers in both the width (W) and length (L) dimensions. Tumor volume was then calculated using the formula (W × W × L)/2. The weights of the mice were taken using a scale that was zeroed prior to each use. Mouse weights were graphed as a proxy to assess toxicity in Appendix A Figure A1.

The following criteria were used to determine when mice should be euthanized in order to comply with IACUC standards: (1) tumor size > 1.5 cm in any dimension, (2) ulceration of tumor through skin surface, (3) weight loss of 20%, (4) signs of physical distress such as hunched posture, lethargy, or inability to ambulate. Termination due to the fourth criterion was not observed in this study. Eleven mice were euthanized prior to the conclusion of the monitoring period. In the control group, 4 mice were euthanized (3 for size and 1 for ulceration). In the 5-FU group, 2 mice were euthanized for tumor size. In the carfilzomib group, 3 mice were euthanized (1 for size and 2 for ulceration). In the combination group that received both 5-FU and carfilzomib, 2 mice were euthanized (1 for size and 1 for weight loss).

### 2.6. RNA Extraction

RNA was extracted from MKN45 cells and human gastric tissue biopsy samples obtained from the University of California San Diego Biorepository, Moores Cancer Center. MKN45 cells grown in T75 flasks were dissociated using trypsin-ethylenediamine tetraacetic acid (EDTA) (Thermo Fisher, Waltham, MA, USA) and centrifuged at 300× *g* for 10 min at 4 °C; the resulting pellet was then homogenized in 1 mL of TRIzol using Direct-zol RNA MiniPrep Kit (Zymo Research, Irvine, CA, USA) following the manufacturer’s instructions and stored at −80 °C until further use. Frozen gastric tissue samples were pulverized using a chilled mortar and pestle, then homogenized in 1 mL of TRIzol reagent (Invitrogen, Carlsbad, CA, USA) with a Dounce homogenizer. RNA was then extracted using Direct-zol RNA MiniPrep Kit (Zymo Research, Irvine, CA, USA), as described in our previous studies [17,18], according to the manufacturer’s instructions.

### 2.7. cDNA Synthesis and Quantitative Real-Time RT-PCR

Gene expression profiling was performed as described in our previous studies [17,18]. A total of 2 µg of RNA isolated from either human tumor samples or MKN45 cells was reverse transcribed into cDNA using the High Capacity cDNA Reverse Transcription Kit (Thermo Fisher, Waltham, MA, USA), as per manufacturer’s instructions. Real-time qRT-PCR (quantitative reverse transcription polymerase chain reaction) was performed on the StepOne Plus Real Time PCR system (Applied Biosystems, Carlsbad, CA, USA) using SYBR Green Supermix (Biorad, Irvine, CA, USA). A total of 1 μL of cDNA was used per well for a 10 μL total reaction mix. The amplification conditions were as follows: initial cycle of 95 °C for 5 min, annealing at 60 °C for 20 s, and extension at 72 °C for 40 s. Expression levels of *PSMB8*, *PSMB9*, *PSMB10*, *CASP1*, *IDO1*, and *MMP13* were normalized to the housekeeping gene hypoxanthine phosphoribosyl transferase 1 (*HPRT1*). All of the above-listed genes were assessed in MKN45 cells and only *PSMB8* expression levels were assessed in patient samples. The data collected were analyzed using comparative cycle threshold (CT) calculations (ΔCT Applied Biosystems). These data were plotted using GraphPad Prism software (v10.5.0, La Jolla, CA, USA). The primers used are listed in Appendix A Table A1.

### 2.8. Paraffin Embedding of Tumor Tissue Samples

MKN45-derived tumors were paraffin embedded according to the following procedures. A tumor tissue sample from each mouse was fixed in 10% neutral buffered formalin, then embedded in paraffin and sectioned on a microtome. Sections of 5 μm were mounted onto glass slides and used for immunohistochemistry (IHC) or TUNEL (terminal nucleotidyl transferase-mediated dUTP-biotin nick end-labelling) assay.

### 2.9. Immunohistochemistry (IHC)

IHC was performed as described in our previous study [15]. Paraffin-embedded gastric tissue slides were deparaffinized in xylene and a decreasing ethanol dilution series, followed by antigen retrieval in citrate buffer heated to 90 °C for 20 min. The sections were then washed in 0.3% Triton X-100 solution and blocked for 1 h with a solution of 3% bovine serum albumin (BSA), 0.1% Tween 20, 0.1% Triton X-100, and 5% normal goat serum in PBS at 22–25 °C. The sections were washed with PBST (0.1% Tween in PBS) and stained with primary antibody (rabbit anti-human Ki67, ab16667; Abcam, Waltham, MA, USA, 1:100 dilution) at 4 °C overnight. This was followed by incubation with an HRP-conjugated anti-rabbit secondary antibody (Cell Signaling, Danvers, MA, USA, cat#7074S, 1:200 dilution) at 25 °C for 1 h. Peroxidase substrate was applied for 5 min, and Harris’ hematoxylin was applied for 2 min. The quantification of positive staining was performed via imaging with the Olympus VS200 Slide Scanner (UCSD School of Medicine Microscopy Core, La Jolla, CA, USA) and analyzed using QuPath software (v8.2.0) [31].

### 2.10. TUNEL Assay

TUNEL assay was performed on paraffin-embedded gastric tumor tissue slides using the ApopTag Peroxidase In Situ Apoptosis Detection Kit (S7101, Sigma Aldrich, St. Louis, MO, USA), as described in our previous study [13] and following manufacturer’s instructions with some modifications, wherein the peroxidase substrate exposure time was decreased to 30 s and methyl green to 5 min. The quantification of positive staining was performed via imaging with the Olympus VS200 Slide Scanner (UCSD School of Medicine Microscopy Core, La Jolla, CA, USA), followed by analysis with QuPath software (v8.2.0) [31].

### 2.11. Statistical Analysis

Statistical analysis was performed using GraphPad Prism (La Jolla, CA, USA). ANOVA with Bonferroni’s correction (for normal distribution) was used for multiple comparisons. *p*-values ˂ 0.05 were considered statistically significant.

## 3. Results

### 3.1. Elevated Expression of DEGs in the Accelerated Murine Gastric Cancer Model

**Figure 3:**
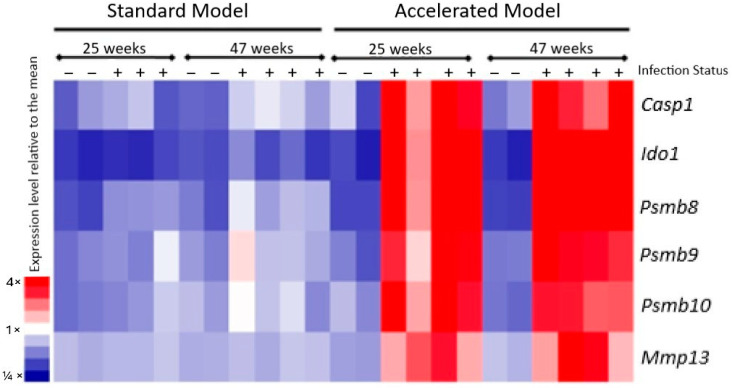
Heatmap showing differential expression of select DEGs in the standard and accelerated models, wherein the standard model is *H. felis*-infected WT mice and the accelerated model is H. felis-infected Myd8^−/−^ mice. Relative expression of *Casp1*, *Ido1*, *Psmb8*, *Psmb9*, *Psmb10*, and *Mmp13* was measured in the standard and accelerated models. For both models, expression of these DEGS was identified by microarray analysis of mouse stomach tissue sections following infection with *H. felis* for 25 weeks and 47 weeks. Each column represents a mouse either infected with *H. felis*, denoted by “+”, or left uninfected, denoted by “−“. As indicated by the color scale on the left, a brighter red color indicates a greater degree of relative expression compared to the mean, while a deeper blue indicates a lesser degree of expression compared to the mean.

Reanalysis of the microarray data collected from the gastric tissue of our accelerated model reveals several GC-induced DEGs, including *Psmb8*, *Psmb9*, *Psmb10*, *Casp1*, *Ido1*, and *Mmp13* [19]. We observed high expression of all these DEGs in the accelerated model at both the 25-week and 47-week time-points compared to the standard model (Figure 3). This increased expression may suggest a potential role for these genes in disease progression, given the rapid disease progression and severe disease pathology observed in the accelerated model [17,18].

### 3.2. Elevated PSMB8 Expression Observed in MKN45 Cells and Gastric Cancer Patient Biopsy Samples

**Figure 4:**
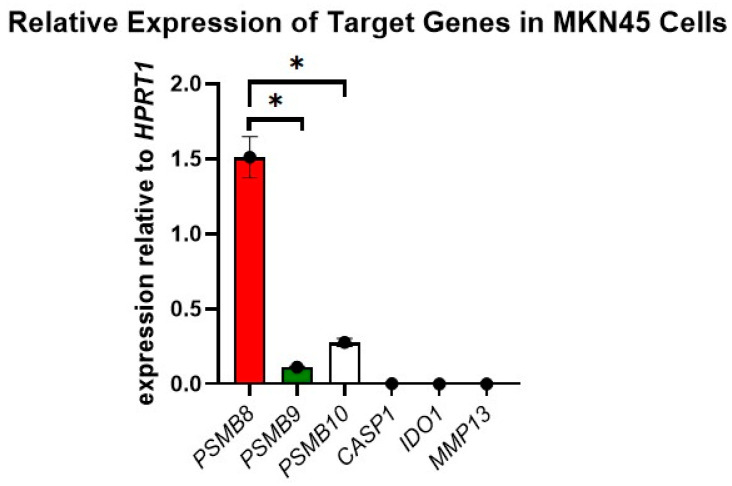
Relative expression of selected DEGs in MKN-45 cells. RT-qPCR was performed with cDNA from MKN45 cells to detect the levels of *PSMB8*, *PSMB9*, *PSMB10*, *CASP1*, *IDO1*, and *MMP13*. Expression of genes was measured relative to the expression of housekeeping gene *HPRT1*. Statistical analysis was performed using Graphpad Prism. Data are presented as mean ± SEM. Asterisks (*) indicate statistically significant differences; *, *p* < 0.01.

**Figure 5:**
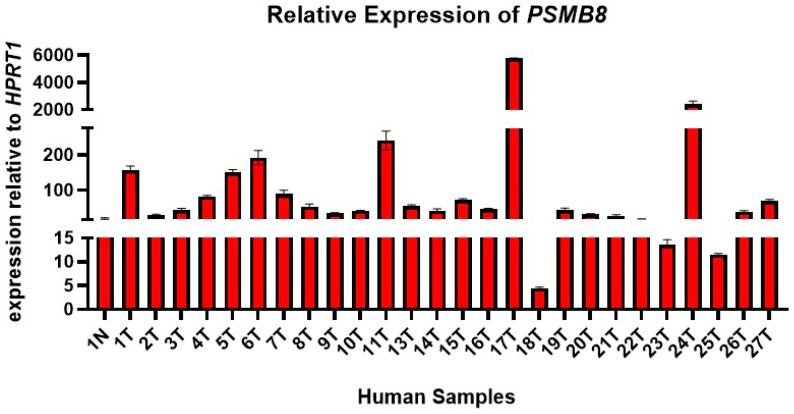
*PSMB8* expression in gastric cancer patient samples. RT-qPCR was performed with cDNA from GC patients (*n* = 26) to detect levels of *PSMB8*. Individual ‘1′ had a paired sample of normal tissue, signified by the ‘N’ at the end of the label. All sample labels ending with ‘T’ indicate a tumor sample. Expression was measured relative to the expression of housekeeping gene *HPRT1*. Statistical analysis was performed using Graphpad Prism. Data are presented as mean ± SEM.

In order to determine whether the DEGs in the accelerated model have a role in disease progression, we first checked the expression levels of these DEGs via qRT-PCR in MKN45 cells. Highly elevated levels of *PSMB8* were observed in comparison to *PSMB9*, *PSMB10*, *CASP1*, *IDO1*, and *MMP13* (Figure 4), indicating its potential influence in disease progression. Furthermore, to validate these findings, we measured the expression levels of *PSMB8* in human GC tissue samples. High expression of *PSMB8* was observed (Figure 5). Thus, confirming the clinical relevance of the study and the potential of *PSMB8* as a drug target candidate.

### 3.3. Carfilzomib Treatment Significantly Slows Tumor Growth

Using a publicly available pharmaceutical database [19], we searched for a drug that specifically targets *PSMB8* and identified carfilzomib as a potential drug candidate. We then evaluated its efficacy as a potential drug for treating GC by monitoring tumor growth, cell proliferation, and apoptosis in our xenograft GC model [32].

**Figure 6:**
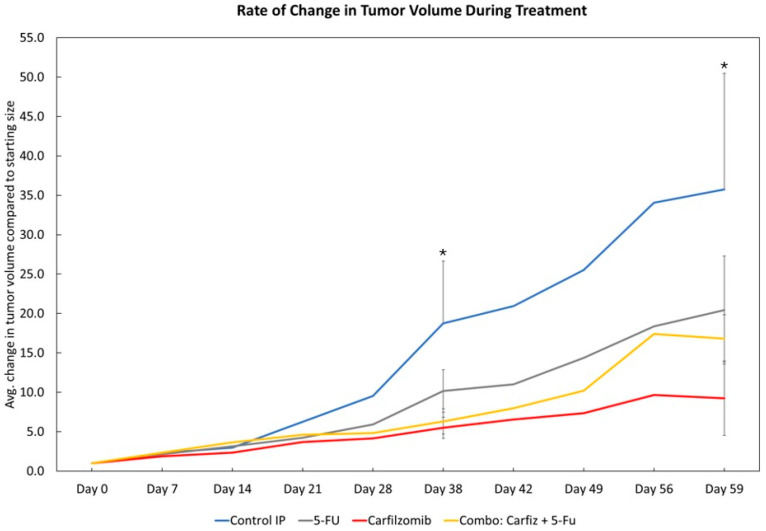
Tumor volume measurements. Rate of increase in tumor volume was measured over the span of 59 days. (Control group *n* = 6; 5FU group *n* = 8; Carfilzomib group *n* = 7; Combination *n* = 8). Statistics performed using Graphpad Prism. Asterisks (*) indicate the statistically significant differences between the data points on the blue line, indicating the progress of control mice, and the data points on the red line, indicating carfilzomib-treated mice. Data are presented as mean ± SEM. *, *p* < 0.05.

**Figure A2:**
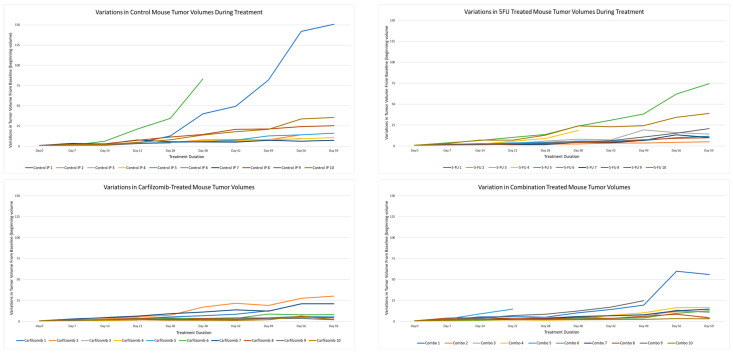
Individual mouse tumor growth curves. Each line on each graph represents the tumor volume of each individual mouse in each treatment group. (**A**) Control group *n* = 10; (**B**) 5FU group *n* = 10; (**C**) Carfilzomib group *n* = 10; (**D**) Combination group *n* = 10.

Tumor volume was monitored over the course of 59 days of treatment. The mice in the control group showed a larger continuous increase in tumor volume over the given time period than those in the treatment groups (the 5FU, carfilzomib, and combination groups). The mice treated with carfilzomib showed a lower rate of tumor growth than the mice in other two treatment groups (Figure 6). In particular, when compared to the control group, the mice treated with carfilzomib exhibited a substantially lower tumor volume than those in the control group, which was significant at two time-points: 38 days (*p* = 0.048) and 59 days (*p* = 0.035) (Figure 6). Individual growth curves are available in Appendix A Figure A2. Within the treatment groups, the carfilzomib treatment group showed a slow increase in tumor volume. These results suggest that carfilzomib retards tumor growth and strengthens its potential for use in the treatment of GC.

### 3.4. Cell Proliferation Is Significantly Impeded by Treatment with Carfilzomib

**Figure 7:**
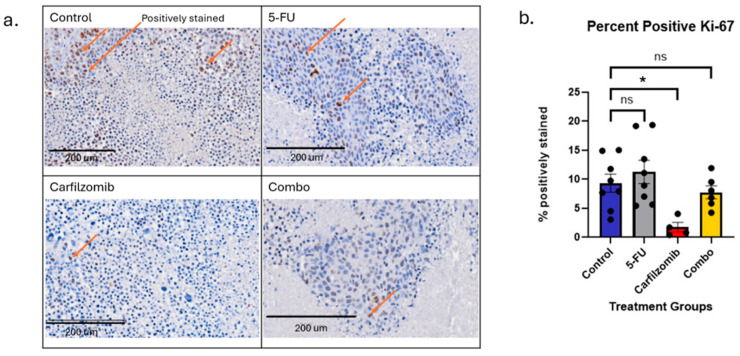
Quantification of Ki67 staining. (**a**) Representative image from each group; orange arrows indicate a positive Ki67 stain. Scale bars represent 200 µm. (**b**) Bar graph depicting the average percentage of positively stained cells in each group. Each black dot represents an individual mouse. Statistics performed using Graphpad Prism; the asterisks (*) indicate statistically significant differences (Control group *n* = 6; 5FU group *n* = 8; Carfilzomib group *n* = 7; Combination *n* = 8). Data are presented as mean ± SEM. *, *p* < 0.01; ‘ns’ = non-significant.

Ki67 staining of tumor cells was performed to quantify the level of cellular proliferation in response to various treatments. Inhibition of tumor cell proliferation is indicative of effective treatment. A marked reduction in positive cells was observed in the carfilzomib treatment group in comparison to the other groups (Figure 7a). We also observed that the carfilzomib-treated group showed a significantly lower percentage of Ki67-positive cells compared to both the control (*p* < 0.01) and 5-FU (*p* < 0.01) groups (Figure 7b). This reduction in cell proliferation is indicative of successful treatment by carfilzomib.

### 3.5. Carfilzomib Induces Apoptosis in Tumor Cells

**Figure 8:**
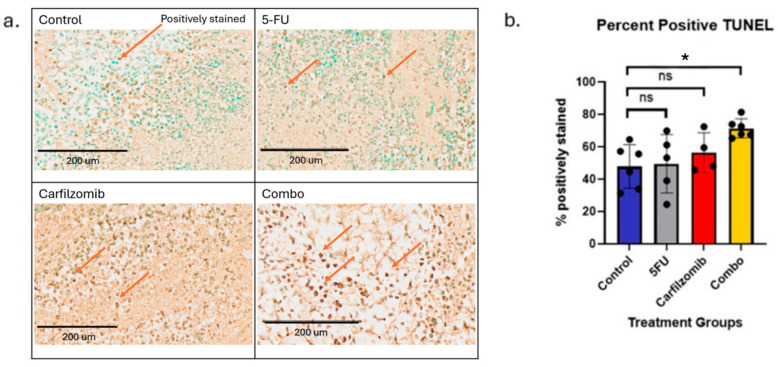
Quantification of TUNEL staining. (**a**) Representative image from each group; orange arrows indicate a positive TUNEL stain. Scale bars represent 200 µm. (**b**) Bar graph depicting the average percentage of positively stained cells in each group Each black dot represents an individual mouse. (Control group *n* = 6; 5FU group *n* = 8; Carfilzomib group *n* = 7; Combination *n* = 8). Statistics performed using Graphpad Prism. The asterisks (*) indicate statistically significant differences. Data are presented as mean ± SEM. *, *p* < 0.01; ‘ns’ = non-significant.

Moreover, in order to determine the mechanism by which carfilzomib slowed tumor growth, we performed the TUNEL assay to measure apoptosis in the different treatment groups. A marked increase in positively stained cells was observed in the carfilzomib and combination treatment groups, as seen in the representative images (Figure 8a). We observed that the combination group showed a significant increase in positive TUNEL staining compared to 5-FU-alone (*p* = 0.027) and control (*p* = 0.003) groups. However, no significant difference was observed in TUNEL staining between the combination and carfilzomib-alone group (Figure 8b). Therefore, this increase in positive TUNEL staining suggests that carfilzomib induces apoptosis in tumor cells. This strongly supports the potential use of carfilzomib in the treatment of GC.

## 4. Discussion

Proteasomes are one of the most important components of the ubiquitin–proteasome system (UPS) for maintaining protein homeostasis in the cell. Immunoproteasomes, an isoform of the proteasome, specialize in processing antigens for presentation via the major histocompatibility complex (MHC) upon stimulation, where the β1, β2, and β5 subunits of the 26S proteasome are substituted by corresponding immune subunits, β1i (*PSMB9*), β2i (*PSMB10*), and β5i (*PSMB8*), to form an immunoproteasome [20,33,34]. Cancer cells rely heavily upon their function for survival and proliferation [20,34,35]. Moreover, elevated expression of immunoproteasomes has been observed in blood cancers such as multiple myeloma, as well as multiple solid tumors including GC [34]. This suggests that immunoproteasomes can serve as targets in treating cancer. Our accelerated model revealed high expression of *Psmb8* [14,15], the β5i subunit of the immunoproteasome [20,35]. We further confirmed expression of *PSMB8* in both the human GC cell line MKN45 and patient gastric biopsy samples via qRT-PCR. The patient samples generally showed heightened expression, despite the variation in patient demographics, disease stage, and treatment history. It is possible that the level of *PSMB* expression may be due to one of the factors reported for other genes [36]; a well-selected patient pool would be required to draw specific conclusions. Nevertheless, what this study suggests is that gastric cancer patients with high expression levels of *PSMB8* in their tumors may benefit from targeted treatment with carfilzomib. Previous studies have shown that *PSMB8* plays an important role in hepatocellular carcinoma through interactions with zinc finger family proteins [37]. More broadly, a pan-cancer analysis found that of 33 cancer types assessed, overexpression of *PSMB8* in certain cancers was associated with poor clinical outcomes [38]. Moreover, upregulated expression of the whole immunoproteasome has been observed in multiple solid tumor cancers, such as non-small cell lung carcinoma and prostate cancer [39,40]. It has also been specifically noted that elevated nuclear expression of *PSMB8* in GC patients is correlated with a decrease in overall patient survival by promoting the migration and invasion of GC cells [41]. These data nonetheless present *PSMB8* as a putative therapeutic target.

Carfilzomib is a proteasome inhibitor that specifically binds to *PSMB8* [35,40]; on the other hand, 5-FU is an inhibitor of DNA and RNA synthesis and a primary component of FLOT, a standard treatment regime for GC patients [29,42]. In this study, we investigated the efficacy of carfilzomib and 5-FU, both as monotherapies and in combination, to understand whether an additive or synergistic effect could be achieved. We observed a significant difference in average tumor volume between the placebo control group and the carfilzomib-treated group. A previous study in a xenograft model showed that oral recombinant methionase (o-rMETase) in combination with 5-FU worked synergistically to suppress tumor growth compared to the monotherapy treatment groups and untreated control [29]. In contrast to these findings, we observed that amongst the treatment groups, the carfilzomib treatment group showed significant retardation in tumor growth. This could be attributed to the fact that both 5-FU and carfilzomib cause cell cycle arrest at different phases of the cell cycle—5-FU at the G1/S phase and carfilzomib at the G2/M phase [43,44]. This conflict of arrest may cause the two drugs to interfere with each other’s mechanisms. Moreover, inhibition of the immuno/proteasome’s function by carfilzomib also affects additional pathways, including the apoptosis, autophagy, and nuclear factor κ-light-chain-enhancer of activated B cells (NF-κB) pathways, as well instigating the unfolded protein response (UPR); this may explain the marked reduction in cell density and the slowing of tumor growth, since this response pathway in particular culminates in apoptosis when homeostasis is not restored due to carfilzomib’s activity [44,45,46]. These results, therefore, suggest that carfilzomib has a robust anti-tumor activity and is a potential drug candidate.

Moreover, it is important to note that we observed a significant difference in tumor growth between the placebo control and carfilzomib treatment groups at day 38 and day 59, the final time point, attributing this to the fact that treatment with carfilzomib limited the growth of the tumor. This implies that treatment with carfilzomib may improve the clinical outcomes for patients with GC, as it may help in retarding tumor growth, thereby allowing an effective surgical resection. Previously, it has been shown that perioperative treatment with FLOT increased patient survival from a median of 35 months to a median of 50 months compared to older therapies [47]. In our study, carfilzomib monotherapy retarded tumor growth more effectively than both the combination treatment and 5-FU monotherapy. These results suggest that perioperative treatment with carfilzomib alone is sufficient for increasing overall patient survival, thereby strengthening the potential of carfilzomib for treating GC.

Inhibition of cell proliferation is an indicator of anti-tumor activity that can be measured by Ki-67 expression. Several studies have associated Ki-67 expression with the effectiveness of chemotherapy in GC [48,49], as previously shown in a study where high expression of Ki-67 was associated with shorter disease-free survival and overall survival in GC patients who received neoadjuvant FLOT chemotherapy [49]. Miyake et al. [29] showed that a combination of 5-FU and o-rMETase led to reduced expression of Ki-67. However, in our study, we observed the highest reduction in Ki-67 expression in the carfilzomib treatment group, which could be attributed to the fact that carfilzomib inhibits the activity of the immunoproteasome, which consequently interferes with the activity of multiple pathways, such as NFκB and p53-p21-RB signaling, leading to inhibition of cell proliferation [45,47]. Therefore, this suggests that carfilzomib alone can effectively inhibit cell proliferation in tumor cells, thus further supporting its use in the treatment of GC.

Apoptosis is a common measure of successful drug treatment, as it triggers effective killing of cancer cells. Previously, Li et al. [50] have shown that treatment with a combination of TNF-related apoptosis inducing ligand (TRAIL) and 5-FU induced significant apoptosis in comparison to monotherapy. However, in this study, we observed higher induction of apoptosis in both the combination and carfilzomib treatment groups in comparison to the other treatment groups. Moreover, only the combination group showed significantly high levels of apoptosis, though the carfilzomib group showed elevated levels that did not reach statistical significance. From this, we can see that carfilzomib does have high anti-tumor activity when administered as a monotherapy in comparison to 5-FU, but also has the ability to enhance the activity of 5-FU when administered in combination. A previous study in lung cancer showed that even though 5-FU caused cell cycle arrest, it also induced the autophagic pathway, through which the cancer cells were able to evade apoptosis [39]. Like 5-FU, carfilzomib also induces autophagy, but the process is hampered by the robust induction of apoptosis via the disruption of Beclin1 and p62 inactivation [51]. Thus, this suggests that carfilzomib not only possesses an anti-tumor activity but also enhances the anti-tumor activity of 5-FU when used in combination and strengthens its potential as a drug candidate.

This study has its own set of limitations. One limitation of this study is that the degree of inhibition of *PSMB8* by carfilzomib was not measured in vivo, such as via an enzyme kinetics assay. Another limitation of the study is that the off-target effects of carfilzomib were not assessed, thus further research is required to assess the off-target effects of carfilzomib. Moreover, as previously mentioned, other studies have noted instances of off-target binding, but those were unlikely to have significant effects at the concentration required for therapeutic effectiveness [28,52]. Nevertheless, this needs to be confirmed in GC applications. Additionally, cardiotoxicity, a consequence of off-target binding, has been noted as a side effect of carfilzomib treatment in multiple myeloma [53,54], and this also needs to be investigated in the context of GC treatment. Finally, the effects of carfilzomib on the tumor microenvironment cannot be inferred in this study due to the nature of xenograft models—which lack an intact immune system—so, no information can be obtained about the coaction of the immune system with the drugs tested here [55,56]. Therefore, future studies need to be carried out using immunocompetent models or patient-derived organoids to learn more about the potential effects of such treatments on the tumor microenvironment. Furthermore, this is a preclinical model with limited extrapolation to patient use and cannot account for patient demographics such as tumor subtype, stage of disease, or other such complexities.

## 5. Conclusions

In conclusion, we suggest that carfilzomib is a strong inhibitor of the immunoproteasome and could serve as a promising drug candidate for targeted therapy in GC and other solid tumors where elevated expression of the immunoproteasome is associated with poor clinical outcomes. Carfilzomib possesses a robust anti-tumor activity as it is able to inhibit tumor growth by inducing tumor cell loss via apoptosis and impeding cell proliferation by inhibiting immunoproteasomal function, consequently leading to induction or inhibition of various pathways like autophagy and NFκB and p53-p21-RB signaling. Thus, the drug shows great potential to be part of a targeted treatment plan for GC, either in combination with the standard treatment plan already in place or as a stand-alone monotherapy that may improve the overall disease-free survival rate in patients with GC. However, further studies need to be carried out to test its efficacy in combination with other drugs, such as dexamethasone, which is already used with carfilzomib in clinical regimens for the treatment of multiple myeloma. Therefore, future clinical evaluation in GC is a requisite to completely assess the true potential of carfilzomib.

## Figures and Tables

**Table 1:** Clinical data of gastric cancer patients.

ID	Patient Sex	Patient Age	Patient Race/Ethnicity	Primary	Metastatic	Stage	Chemotherapy
1T/1N	F	53	Asian	Adenocarcinoma, signet ring-cell	No	IIA (ypT3ypN0)	EOX
2T	F	25	Hispanic	Adenocarcinoma, diffuse type	yes	IV (ypT4bypN3bypM1)	EOX/FOLFIRI
3T	M	51	White	Adenocarcinoma	no	IIB (ypT4aN0)	Yes, unspecified
4T	M	78	White	Invasive adenocarcinoma	yes	IIIC (pT4aN3a)	No
5T	F	49	White	invasive adenocarcinoma, signet ring	yes	IIB (pT4aN0)	No
6T	F	48	Asian	adenocarcinoma, diffuse type. Signet-ring	no	IIIC (pT4aN3a)	No
7T	F	77	Asian	gastric adenocarcinoma	no	yT3N1	FOLFOX (neo-adjuvant)
8T	F	81	Vietnamese	gastric adenocarcinoma, intestinal type	invades serosa	pT4aN0	No
9T	M	45	White	signet ring gastric adenocarcinoma	yes	pT4aN3bM1	No
10T	F	66	Asian	gastric adenocarcinoma, diffuse type with signet ring	no	ypT4aN0	FLOT
11T	M	81	Asian	Gastric adenocarcinoma	yes	ypT3N3a	FOLFOX
13T	M	56	Other Hispanic, Latino, or Spanish Origin	neoplastic	N/A	G3 mpT4a N3a M1	No
14T	M	69	Other Hispanic, Latino, or Spanish Origin	neoplastic	N/A	stage IV, ypT4b N3b M1	Yes, unspecified.
15T	M	32	Other Hispanic, Latino, or Spanish Origin	Neoplastic, mucinous adenocarcinoma with signet ring cell features	N/A	ypT4aN1	Yes, unspecified.
16T	F	70	Asian	adenocarcinoma	N/A	stage IIB, pT3N1	No
17T	M	73	Asian	Invasive poorly differentiated adenocarcinoma with focal signet ring cell features	N/A	Stage IIA, T3N0M0G3	Yes, unspecified.

N/A indicates that data were not available for a given sample within the designated category.

## Data Availability

The original contributions presented in this study are included in the article. Further inquiries can be directed to the corresponding author.

## Appendix A

**Table A1:** Primers used for qRT-PCR. All sequences listed are 5′- > 3′ and for use on human samples.

Gene	Forward Primer	Reverse Primer
*PSMB8*	CCTTACCTGCTTGGCACCATGT	TTGGAGGCTGCCGACACTGAAA
*PSMB9*	CGAGAGGACTTGTCTGCACATC	CACCAATGGCAAAAGGCTGTCG
*PSMB10*	GGACAAGAGCTGCGAGAAGATC	ATCTTGGACGCCACCATCCGTG
*CASP1*	TCCAATAATGGACAAGTCAAGCC	GCTGTACCCCAGATTTTGTAGCA
*IDO1*	GCCTGATCTCATAGAGTCTGG	TGCATCCCAGAACTAGACGTG
*MMP13*	TGACTATGCGTGGCTGGAA	AAGCTGAAATCTTGCCTTGGA
*HPRT1*	CCTGGCGTCGTGATTAGTGAT	AGACGTTCAGTCCTGTCCATAA
